# Biological and Pathological Implications of an Alternative ATP-Powered Proteasomal Assembly With Cdc48 and the 20S Peptidase

**DOI:** 10.3389/fmolb.2018.00056

**Published:** 2018-06-08

**Authors:** Masatoshi Esaki, Ai Johjima-Murata, Md. Tanvir Islam, Teru Ogura

**Affiliations:** ^1^Department of Molecular Cell Biology, Institute of Molecular Embryology and Genetics, Kumamoto University, Kumamoto, Japan; ^2^Core Research for Evolutional Science and Technology, Japan Science and Technology Agency, Saitama, Japan; ^3^Program for Leading Graduate Schools “HIGO Program, ” Kumamoto University, Kumamoto, Japan

**Keywords:** AAA ATPase, Cdc48/p97/VCP, proteasome, proteolysis, Sod1, ALS

## Abstract

The ATP-powered protein degradation machinery plays essential roles in maintaining protein homeostasis in all organisms. Robust proteolytic activities are typically sequestered within protein complexes to avoid the fatal removal of essential proteins. Because the openings of proteolytic chambers are narrow, substrate proteins must undergo unfolding. AAA superfamily proteins (ATPases associated with diverse cellular activities) are mostly located at these openings and regulate protein degradation appropriately. The 26S proteasome, comprising 20S peptidase and 19S regulatory particles, is the major ATP-powered protein degradation machinery in eukaryotes. The 19S particles are composed of six AAA proteins and 13 regulatory proteins, and bind to both ends of a barrel-shaped proteolytic chamber formed by the 20S peptidase. Several recent studies have reported that another AAA protein, Cdc48, can replace the 19S particles to form an alternative ATP-powered proteasomal complex, i.e., the Cdc48-20S proteasome. This review focuses on our current knowledge of this alternative proteasome and its possible linkage to amyotrophic lateral sclerosis.

## The ubiquitin- and proteasome-dependent degradation pathway

**Figure 1:**
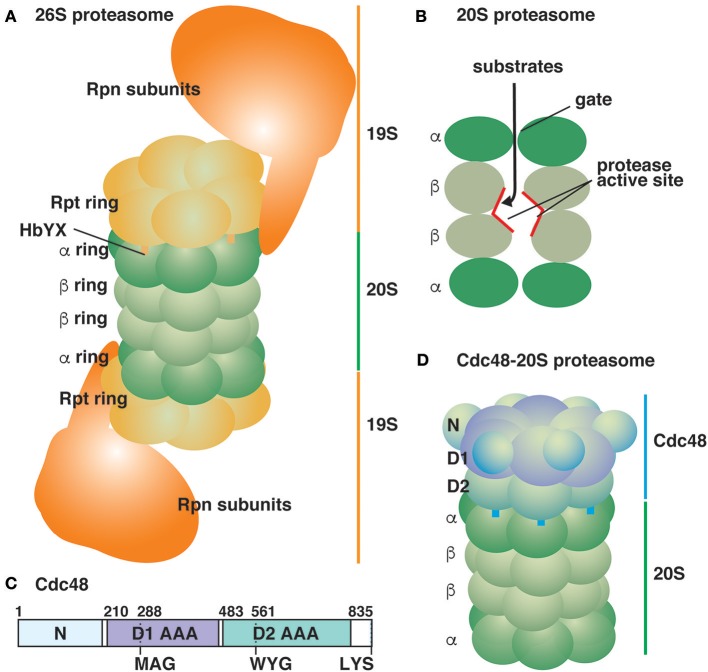
Schematic representation of ATP-powered proteolysis machines in the eukaryotic cytosol. **(A)** The typical 26S proteasome is composed of the 20S proteasome sandwiched between two 19S RP (Huang et al., 2016; Schweitzer et al., 2016). Thirteen Rpn subunits of 19S RP are depicted as a large mass for simplicity. **(B)** A lateral cutaway view of the 20S proteasome. The proteolysis sites are located inside the 20S proteasomal chamber. **(C)** Yeast Cdc48 is an 835-amino acid residue protein composed of two AAA domains (D1 and D2). The pore loops of the D1 and D2 AAA domains are shown. The protein ends with the sequence LYS. **(D)** The Cdc48-20S proteasome (Davies et al., 2008; Barthelme et al., 2014).

The eukaryotic 26S proteasome is a robust protein degradation machine that is conserved in all eukaryotes. The 26S proteasome is composed of a 20S core peptidase, which is also called as the 20S proteasome, and 19S regulatory particles (RP) (Figure 1A; for review, see Baumeister et al., 1998; Finley, 2009; Tanaka, 2009; Finley et al., 2016). The 20S proteasome possesses two copies of 14 different protein subunits, which are assembled into two sets of two heptameric rings, named α and β. Two β rings are sandwiched between two α rings, and the four tandem rings form a barrel-shaped structure. Proteolytic activities occur in the central cavity of the β rings as three β subunits possess caspase-, chymotrypsin-, and trypsin-like proteolytic activities, respectively (Figure 1B). The α rings cap both sides of the cavity formed by the β rings and constitute a narrow channel with a gate to restrict the entry of substrate proteins into the proteolytic sites. 19S RP are composed of six homologous, but distinct AAA proteins (Rpt subunits), which form a hexameric ring structure, and 13 non-ATPase subunits (Rpn subunits). The AAA hexameric ring facilitates protein unfolding by passing the substrates through the central pore to traverse the narrow entrance of the α ring in the 20S proteasome. The C-terminal HbYX (hydrophobic, tyrosine, and any amino acid residues) sequence motif of the Rpt subunits is thought to dock into conserved pockets on the outer surface of the α rings, which may open the gate (Figure 1A; Smith et al., 2007; Huang et al., 2016; Schweitzer et al., 2016). The Rpn subunits are responsible for substrate specificity and processing.

Most substrate proteins of the 26S proteasome are marked with a small protein called ubiquitin. The C-terminal end of ubiquitin is covalently attached to an amino group of the target protein by an array of ubiquitylation enzymes. The attached ubiquitin molecules are often modified further with another ubiquitin molecule through the N-terminus or their seven lysine residues. Lysine 48-linked polyubiquitin chains containing 4 or more ubiquitin molecules are recognized by ubiquitin receptors on 19S RP to signal the degradation of client proteins by the 26S proteasome (Thrower et al., 2000). An unstructured region is also required for efficient degradation and interestingly, the polyubiquitin chain signal can work in trans, i.e., a protein with an unstructured region in a protein complex is preferentially degraded even if a polyubiquitin chain is attached to another subunit within the complex (Prakash et al., 2004, 2009). The attached polyubiquitin chains are cleaved off by deubiquitylation enzymes in 19S RP prior to degradation.

## CDC48 as a segregase of polyubiquitylated proteins

The AAA protein Cdc48, also known as p97, VCP, and TER94, is one of the most abundant intracellular proteins and is highly conserved in all eukaryotic species. Cdc48 is located in the cytosol and nucleus, and plays essential roles in many cellular processes including the endoplasmic reticulum (ER)- and mitochondria-associated protein quality control systems, organelle membrane fusion, transcription activation, DNA replication, meiosis progression, and prevention of protein aggregation, which have been extensively summarized recently (Bodnar and Rapoport, 2017b; Esaki, 2017; Hänzelmann and Schindelin, 2017; Saffert et al., 2017; Stach and Freemont, 2017; Ye et al., 2017; van den Boom and Meyer, 2018). The major function of Cdc48 is to segregate substrate proteins from protein complexes and organelle membranes, and these liberated proteins are then transferred to the degradation machinery or refolded for recycling. In the ubiquitin and 26S proteasome system, Cdc48 functions upstream of proteasomal degradation, though the 26S proteasome also has the ability to degrade model polyubiquitylated substrate proteins independently of Cdc48 (Thrower et al., 2000). A comprehensive analysis has indicated that Cdc48 and its cofactor Ufd1-Npl4 complex predominantly recognize K48-linked polyubiquitin chains (Tsuchiya et al., 2017). After processing by the Cdc48-Ufd1-Npl4 complex, K48-linked polyubiquitylated substrate proteins are transferred to a shuttle factor, Rad23, Dsk2, or Ddi1, and then delivered to the 26S proteasome for degradation. It is most likely that some, if not all, ubiquitin molecules are removed upon processing by Cdc48 because deubiquitylation enzymes such as Otu1/YOD1 (Rumpf and Jentsch, 2006; Ernst et al., 2009) and Ubp3 (Ossareh-Nazari et al., 2010) bind to Cdc48 and are required for Cdc48-mediated reactions. Therefore, after processing by Cdc48, K48-linked polyubiquitin chains are recaptured by a ubiquitin ligase Ufd2 (Richly et al., 2005).

The mechanism by which Cdc48 segregates substrate proteins has been a matter of debate for a long time. Cdc48 is composed of an N-terminal regulatory domain followed by two AAA domains (Figure 1C). Cdc48 cofactors such as the Ufd1-Npl4 heterodimer bind to the N-terminal domain and the C-terminal portion of Cdc48. The two AAA domains form two stacked hexameric rings sharing a connected central pore (Figure 1D; DeLaBarre and Brunger, 2003). A threading mechanism has been proposed as a common molecular mechanism for several AAA hexameric rings including the Rpt subunits of 19S RP (Ogura et al., 2008). In this mechanism, substrate proteins pass through the narrow central pore of AAA domains in an ATP-dependent manner, resulting in the unfolding of substrate proteins. Genetic and biochemical analyses for Cdc48 suggest that the central pore as well as ATPase activity of the AAA domains are essential (DeLaBarre et al., 2006; Esaki and Ogura, 2010; Esaki et al., 2017). Recent approaches using purified components have directly tested the threading mechanism of Cdc48 in detail (Blythe et al., 2017; Bodnar and Rapoport, 2017a). Two groups constructed K48-linked polyubiquitylated fluorescent proteins as model substrates, and independently demonstrated that the Cdc48-Ufd1-Npl4 complex facilitated the unfolding of these fluorescent proteins. Bodnar and Rapoport (2017a) also showed that the fluorescent proteins were crosslinked with some residues lining the central pore of Cdc48 in an ATPase-dependent manner, suggesting that Cdc48 also functions based on the threading mechanism. However, the *in vitro* threading efficiency of Cdc48 seems to be low because excess amounts of Cdc48 over the substrates were required for efficient unfolding. Therefore, other molecular mechanisms in addition to the widely-applicable threading mechanism may be involved.

## An alternative ATP-powered proteasome composed of CDC48 and the 20S proteasome

One of essential function of 19S RP is gate-opening for the 20S proteasome, and the C-terminal HbYX sequence motif of the Rpt subunits in 19S RP, which dock into the pockets on the α ring surface, is the key. Short peptides ending with LYR were sufficient to unlock the gate of the 20S proteasome (Smith et al., 2007). Interestingly, most Cdc48 homologs ranging from yeast to humans possess the LYX sequence in their C-termini (Barthelme and Sauer, 2013). Barthelme and Sauer demonstrated that Cdc48 activated the peptidase activity of the 20S proteasome, suggesting that it is capable of unlocking the gates of the proteasomal chamber (Figure 1D; Barthelme and Sauer, 2012, 2013). The activation required ATP binding to either or both of two AAA domains of Cdc48 along with the conserved tyrosine residue in the HbYX motif. Although the functional interaction between purified Cdc48 and the 20S proteasome was described, their physical interaction seems quite transient and is not readily detected without any artificial stabilization such as chemical crosslinking. Electron microscopy of a crosslinked Cdc48-20S proteasome complex revealed that the central pore of Cdc48 is in line with the gate of the proteasomal chamber, suggesting that substrate proteins may pass through the Cdc48 pore and reach the proteolysis site in the 20S proteasome (Barthelme et al., 2014). However, the Cdc48-20S proteasome exhibited no *in vitro* degradation activity for a model folded substrate protein because Cdc48 alone exhibited intrinsically low unfolding activity (Rothballer et al., 2007; Barthelme and Sauer, 2012). The typical threading mechanism-driven unfolding reaction requires a loop structure protruding into the central pore of the AAA domains (pore loop) with a conserved ΦXG (aromatic, any, and glycine residues) sequence motif. Notably, the first aromatic residue has been shown to directly interact with substrate proteins, and conformational changes in the pore loops during the cycle of ATP hydrolysis drive the substrate threading (Schlieker et al., 2004; Ripstein et al., 2017). The ΦXG sequence motif is conserved in the pore loop of the second AAA domain, but not in the first AAA domain, of Cdc48 (Figure 1C; Rothballer et al., 2007; Esaki et al., 2017). Cdc48 mutants with an essential ΦXG sequence motif in the first AAA domain acquired robust unfolding activity *in vitro* although the N-terminal regulatory domain must be removed concomitantly (Rothballer et al., 2007). The hyperactive Cdc48 mutants, together with the 20S proteasome, exhibited efficient degradation of a model folded substrate (Barthelme and Sauer, 2012, 2013).

The *in vitro* functional and physical formation of the Cdc48-20S proteasome was further verified by genetic analysis using the budding yeast *Saccharomyces cerevisiae* (Esaki et al., 2017). Introduction of a ΦXG sequence motif in the first AAA domain of Cdc48 was lethal. This lethal hyperactive mutation could be suppressed by additional mutations preventing the interaction between Cdc48 and the 20S proteasome, suggesting that fatal protein degradation by the hyperactive Cdc48-20S proteasome could account for the lethal phenotype. A copper-zinc superoxide dismutase, Sod1, was found to be degraded upon overexpression of the hyperactive Cdc48 mutant (Esaki et al., 2017). However, Sod1 is known to be one of the most stable proteins (Rakhit and Chakrabartty, 2006), which suggests that Sod1 is an endogenous substrate of the Cdc48-20S proteasome (Esaki et al., 2017).

## Possible linkage of the alternative proteasome to amyotrophic lateral sclerosis

Sod1 is a ubiquitously expressed and highly conserved protein. Sod1 is a homo-dimeric enzyme located mostly in the cytosol, whereas a small proportion was reportedly found in the mitochondrial inter-membrane space (Sturtz et al., 2001). Sod1 detoxifies the superoxide radical to oxygen and hydrogen peroxide (Bermingham-McDonogh et al., 1988). The human Sod1 homolog has been identified as the first and major causative gene for autosomal dominant familial cases of amyotrophic lateral sclerosis (ALS), a fatal neurodegenerative disorder, and some sporadic ALS patients (~3%) also possess mutations in the *SOD1* gene (Chen et al., 2013; Saccon et al., 2013). Over 150 mutations in the *SOD1* gene have been identified from ALS patients, most of which lead to decreased dismutase activity. Loss of dismutase activity in SOD1 by mutations and/or aggregation of mutant proteins was initially thought to cause the ALS pathology (Deng et al., 1993, 2006). However, dismutase activity in transgenic mice, zebrafish, and *Drosophila* with ALS-related SOD1 mutations that caused ALS symptoms, ranged from 0 to 14-fold higher than that in the wild type (Joyce et al., 2011). Moreover, mice and yeasts lacking the *SOD1* gene showed no obvious ALS-associated phenotype (Reaume et al., 1996; Bastow et al., 2016). These observations suggest that a toxic gain-of-function, rather than loss-of-function, in SOD1 mutants triggers the ALS symptoms.

ALS-related Sod1 mutations decreased the protein stability and growth rates of yeast cells (Bastow et al., 2016). Interestingly, the expression of truncated versions of Sod1 in yeast elicited growth retardation without the formation of protein aggregates, suggesting the involvement of possible Sod1 degradation products in ALS. Although rare, a few causative mutations in the human Cdc48 homolog, VCP, were identified in familial ALS patients (Johnson et al., 2010; Tang and Xia, 2016). Most of the ALS-mutations were found in the N-terminal domain of VCP, where many mutations were reported from another VCP-involving fatal neurodegenerative disorder called inclusion body myopathy with Paget's disease of bone and frontotemporal dementia (IBMPFD). An ALS-related mutation, D592N, was found in a loop structure located at the bottom of the central pore of the second AAA domain of VCP (Davies et al., 2008; Johnson et al., 2010). This loop structure was shown to be responsible for the efficient interaction between Cdc48 and the 20S proteasome (Barthelme and Sauer, 2013). Although the D592N mutation affected neither ATPase activity nor the unfolding activity of Cdc48, the degradation activity of the mutant Cdc48-20S proteasome was remarkably reduced (Barthelme et al., 2015). These observations imply that the Cdc48-20S proteasome may possibly be linked to ALS via degradation of SOD1, which could be tested in the future.

In the canonical protein degradation pathway by the 26S proteasome, K48-linked polyubiquitylation marks the client proteins, and is required for recognition by the Cdc48-Ufd1-Npl4 complex. Although polyubiquitylation on ALS-related Sod1 mutants has been reported (Urushitani et al., 2002), it is not clear whether the polyubiquitylation or other features, which would be embedded in its primary sequence or its folded conformation, function as a signal to the Cdc48-20S proteasome. Possible involvements of the Ufd1-Npl4 or other Cdc48 cofactors in the Cdc48-20S proteasome and ALS symptoms remain elusive. *In vitro* reconstitution of Sod1 degradation reaction by the Cdc48-20S proteasome will help our understanding of the molecular determinants of its substrates, the recognition mechanism, and the required factors and features in more detail.

## Perspectives

The 26S proteasome typically contains two 19S RP units which cap both ends of the 20S proteasome (Figure 1A). Eukaryotes additionally contain nonessential and non-ATPase complexes, PA28 and PA200, for activating the 20S proteasome, which could enhance the peptidase activity, but do not support the degradation of intact proteins (Table 1) (Chu-Ping et al., 1992; Dubiel et al., 1992; Murata et al., 2001; Ustrell et al., 2002; Schmidt et al., 2005; Schmidt and Finley, 2014). Structural analyses by electron microscopy showed that two PA28 complexes can simultaneously bind both ends of the 20S proteasome, in a manner similar to that of PA200 (Hendil et al., 1998; Schmidt et al., 2005). In addition, heterologous complexes in which 19S RP binds to one end of the 20S proteasome and PA28 or PA200 binds to the other end were also observed. The Cdc48-20S proteasome complexes visualized by electron microscopy (Barthelme et al., 2014) showed only one Cdc48 bound to either end of the 20S proteasome (Figure 1D; Table 1), probably because of the transient interaction between them. It would be interesting to reveal the possible complexes formed with Cdc48 on both ends of the 20S proteasome and the heterologous complexes with the 20S proteasome sandwiched between Cdc48 and other activators. A proteomic analysis of the budding yeast revealed that the average molar amounts of the Rpt proteins of 19S RP and those of Blm10, the yeast PA200 homolog, are one third of and just a few percent of the 20S proteasome, respectively (Table 1; Kulak et al., 2014). Because the budding yeast lacks PA28 homologs, significant populations of the 20S proteasome are present in the free form or as a complex with Cdc48. It would also be interesting to capture the physical interaction of Cdc48 and the 20S proteasome *in vivo*. Furthermore, recent advances on cryo-electron microscopy will contribute to reveal the molecular architecture of the Cdc48-20S proteasome *in situ* (Huang et al., 2016; Schweitzer et al., 2016; Wehmer and Sakata, 2016; Wehmer et al., 2017).

**Table 1:** Subtypes of proteasome activators in eukaryotes.

**Proteasome activators**	Relative abundancy*	**ATPase activity**	**Unfolding activity**	**Complex with 20S**		
				**Single cap**	**Both caps**	**Hetero caps**
19S RP	~ 40%	+	+	+	+	+
PA200/Blm10	~ 3%	–	–	+	+	+
PA28	N.A.**	–	–	+	+	+
Cdc48	~ 34%	+	+	+	N.D.***	N.D.***

* Relative complex abundancy to that of the 20S proteasome in the budding yeast S. cerevisiae (Kulak et al., 2014)

** not applicable

*** *not determined*.

What is the biological significance of the Cdc48-20S proteasome? Remarkably, Cdc48 itself is an essential protein, but the Cdc48-20S proteasome is not essential for the vegetative growth of yeast cells because removal of the HbYX sequence motif from Cdc48 revealed no obvious growth defects (Esaki et al., 2017). Thus, the essential functions of Cdc48 are independent of the Cdc48-20S proteasome and may involve segregation of client proteins from protein complexes and organelles for further degradation by the 26S proteasome or for recycling. Nevertheless, the lethal phenotype caused by the hyperactive Cdc48 mutants relies on the interaction between Cdc48 and the 20S proteasome (Esaki et al., 2017), implying that the Cdc48-20S proteasome has some nonessential functions *in vivo*. Replacement of the tyrosine residue with a glutamate residue in the C-terminal HbYX motif of Cdc48, which was expected to disrupt efficient complex formation of the Cdc48-20S proteasome, resulted in a hypersensitive growth phenotype in media containing the arginine analog canavanine or oleic acid (Böhm et al., 2011) and led to Cdc48 accumulation in the nucleus (Madeo et al., 1998). Although the mutations also affected the interaction of Cdc48 with some cofactors, these observations suggest that the cytosolic Cdc48-20S proteasome may be critical under certain conditions. This is consistent with the result that the lethality caused by the hyperactive Cdc48-20S proteasome largely depended on cytosolic Cdc48 (Esaki et al., 2017). Conversely, nuclear-localized Cdc48 also plays an important role because elimination of Cdc48 from the nucleus by mutating the nuclear localization signal leads to growth defects (Madeo et al., 1998). IBMPFD-causing mutations suppressed the nuclear entry of VCP (Song et al., 2015). Anchoring of the 26S proteasome to the plasma membrane, which eliminated the 26S proteasome from the nucleus, resulted in a lethal phenotype (Tsuchiya et al., 2013). These observations imply that regulated proteolysis in the nucleus is vital for health and that the Cdc48-20S proteasome may also play an important role in the nucleus. Identification of more endogenous substrates is surely required to understand the biological significance of the Cdc48-20S proteasome in more detail.

The alternative ATP-powered proteasome has just emerged recently. In the canonical pathway, Cdc48-treated substrates are transferred to a shuttling factor followed by processing by 19S RP of the 26S proteasome. Alternatively, Cdc48-treated substrates are directly transported to the 20S proteasomal chamber. A comparative analysis between the endogenous substrates of the Cdc48-20S proteasome will identify the general determinants of their fate.

## Author contributions

ME, MI, and AJ-M performed the research. AJ-M and ME prepared the figure. ME and TO prepared the text.

### Conflict of interest statement

The authors declare that the research was conducted in the absence of any commercial or financial relationships that could be construed as a potential conflict of interest.
